# Altered sensorimotor neuroplasticity in subacute patients with non-severe burn injury

**DOI:** 10.1093/burnst/tkag034

**Published:** 2026-04-30

**Authors:** Grant Rowe, Mark Fear, Dale W Edgar, Aleksandra Miljevic, Tyler Osborne, Natalie Morellini, Merrilee Needham, Ann-Maree Vallence, Fiona Wood

**Affiliations:** School of Psychology, College of Health and Education, Murdoch University, 90 South Street, Murdoch, Perth, WA, Australia; Fiona Wood Foundation, Perth, Australia; Burn Injury Research Unit, The University of Western Australia, Perth, WA, Australia; Fiona Wood Foundation, Perth, Australia; Burn Injury Research Unit, The University of Western Australia, Perth, WA, Australia; Burn Injury Research Node, The Institute for Health Research, The University of Notre Dame, WA, Australia; State Adult Burn Unit, Fiona Stanley Hospital, Perth, Australia; Fiona Wood Foundation, Perth, Australia; Perron Institute for Neurological and Translational Science, Perth, WA, Australia; Fiona Wood Foundation, Perth, Australia; School of Allied Health, College of Health and Education, Murdoch University, Perth, WA, Australia; School of Medical and Health Sciences, Edith Cowan University, Perth, WA, Australia; Fiona Wood Foundation, Perth, Australia; State Adult Burn Unit, Fiona Stanley Hospital, Perth, Australia; Fiona Wood Foundation, Perth, Australia; Perron Institute for Neurological and Translational Science, Perth, WA, Australia; South Metropolitan Health Service, Perth, WA, Australia; The University of Notre Dame, WA, Australia; Murdoch University, Perth, WA, Australia; School of Psychology, College of Health and Education, Murdoch University, 90 South Street, Murdoch, Perth, WA, Australia; Centre for Healthy Ageing, Health Futures Institute, Murdoch University, Perth, WA, Australia; Personalised Medicine Centre, Health Futures Institute, Murdoch University, Perth, WA, Australia; Fiona Wood Foundation, Perth, Australia; Burn Injury Research Unit, The University of Western Australia, Perth, WA, Australia; State Adult Burn Unit, Fiona Stanley Hospital, Perth, Australia; South Metropolitan Health Service, Perth, WA, Australia

**Keywords:** Burn injury, Subacute, Motor performance, Neuroplasticity, Intracortical inhibition

## Abstract

**Background:**

Burn injuries affect the central nervous system, and evidence suggests they induce changes in cortical excitability related to motor dysfunction. The current study investigated markers of neuroplasticity in the motor cortex and motor performance in the subacute period following burn injury.

**Methods:**

Thirty-four patients with a non-severe burn injury (total body surface area: 1.0 ± 2.0%; 16 females; age: 46.0 ± 14.0 years) participated in a longitudinal study with assessments ranging from ~1 to ~17 weeks post-injury. Transcranial magnetic stimulation was used to evaluate motor cortex excitability and short- and long-intracortical inhibition (SICI; LICI) before and after application of paired associative stimulation (PAS) to induce neuroplasticity. Motor performance was assessed using the Purdue Pegboard.

**Results:**

Patients showed improvements in bilateral motor performance from ~3 to ~6 weeks post-injury. PAS did not induce any change in net motor cortex excitability or SICI but increased LICI, which was greatest ~6 weeks post-injury. Adjusted modeling showed that greater PAS-induced increase in LICI was associated with better bilateral motor performance, suggesting an association between PAS-induced neuroplasticity of long-acting inhibitory circuits in the primary motor cortex and motor function following burn injury.

**Conclusions:**

The data provide a neurophysiological basis to test whether targeting inhibitory networks in the subacute period after burn injury can enhance or expedite functional recovery.

## Highlights

- Subacute burn patients showed improvements in bilateral motor performance from ~3 to ~6 weeks post-injury.
- Paired associative stimulation (PAS) did not significantly change net motor cortex excitability.
- PAS did significantly increase GABA_B_-mediated inhibition, with the greatest change early following burn injury.
- Greater PAS-induced increases in long-intracortical inhibition were associated with better bilateral motor performance.

## Background

Burn injuries cause extensive tissue damage, affecting not only the skin but also the peripheral neural architecture. Such changes can result in motor and sensory impairments that significantly diminish quality of life [1], which have been reported in both severe [>20% total body surface area (TBSA)] [2] and non-severe burns (<20% TBSA) [3]. Sensory and motor impairments following burn injury are likely mediated by multiple, interacting factors, including systemic inflammation [4, 5], sensory dysfunction [6, 7], and pain [8, 9], all of which may influence brain regions involved in motor control and affect functional outcomes [10]. While much research has explored the functional consequences of burn injuries, increasing attention is now being directed toward the mechanisms underlying these impairments. Two recent reviews have proposed central nervous system changes, including brain changes, might contribute to persistent functional impairments following burn injury [11, 12]. Neuroplasticity is the brain’s capacity to adapt in response to environmental stimuli and physiological change [13]. This contemporary shift in understanding the mechanisms mediating functional impairments following burn injury is important as it could provide novel therapeutic targets for interventions to enhance recovery [11, 12].

Transcranial magnetic stimulation (TMS) can be used to both measure and induce neuroplasticity [14, 15]. A current generated in the brain via electromagnetic induction depolarizes neurons within a targeted cortical region [14]. When a suprathreshold TMS pulse is applied to the primary motor cortex (M1), it produces a motor evoked potential (MEP), which can be recorded using electromyography (EMG) of the target muscle [15, 16]. The peak-to-peak MEP amplitude provides a marker of corticospinal excitability, and changes in MEP amplitude indicate neuroplasticity [17, 18].

Cortical inhibition plays and important role in neuroplasticity induction [19]. Single- and paired-pulse TMS can be used to measure inhibition in the motor cortex. Single-pulse TMS can be used to measure the cortical silent period (cSP): a period of inactivity in the EMG from a voluntarily contracted muscle following a suprathreshold TMS pulse [20, 21]. The early part of the cSP (<50 ms) is primarily mediated by spinal mechanisms and the later part (50–200 ms) is mediated by cortical mechanisms [22]. The cSP is mediated by GABAergic inhibition, with both GABA_A_ and GABA_B_ receptor activity possibly playing a role [23–27]. Paired-pulse TMS can be used to measure short-interval intracortical inhibition (SICI) [28, 29], which is mediated by GABA_A_ receptor activity [30, 31]: when a subthreshold conditioning stimulus (CS) precedes a suprathreshold test stimulus (TS) by 1–5 ms, short latency and short-lasting inhibition of MEP amplitude is observed.

Recently, TMS has been used to investigate neurophysiological changes in the motor cortex following burn injury. Garside *et al.* [32] reported no overall difference in cSP duration between burn patients ~6 weeks to 8 years after injury and non-injured controls. However, exploratory subgroup analyses showed shorter cSP durations on the burn-affected side of upper-limb burn patients, within 2 years post-injury, with burns <10% TBSA, and those with partial-thickness burns. This suggests non-severe burn injuries may reduce intracortical inhibition. More recent work showed greater SICI (and long-interval intracortical inhibition: LICI) was significantly associated with better bilateral motor performance on the Purdue Pegboard task in burn patients 1–3 years post-injury [33], suggesting intracortical inhibition may influence functional recovery following burn injury. Previous work showed SICI was not different between subacute burn patients (6–12 weeks post-injury) and controls, suggesting that intracortical inhibition may not be altered within this early subacute phase [34]. However, it is possible that changes in intracortical inhibition occur before 6 weeks following burn injury when physiological disruption is greatest [35]. Together, these findings underscore the importance of early assessment to identify neuroplastic changes that may influence functional recovery trajectories in subacute burn patients.

When applied repetitively, TMS can induce neuroplasticity through long-term potentiation- (LTP) and long-term depression (LTD)-like mechanisms [36–38]. Repetitive TMS (rTMS) produces lasting, bidirectional changes in MEP amplitude depending on stimulation frequency and pattern, which are mediated by NMDA-receptor activity [39, 40]. Recent work examined whether rTMS-induced neuroplasticity was associated with functional outcomes following burn injury. Whife *et al.* [41] showed greater neuroplasticity induced by spaced continuous theta burst stimulation (cTBS) in subacute burn patients 12 weeks post-burn was significantly associated with better general health outcomes [measured on the short form-36 (SF-36) survey], suggesting that M1 neuroplasticity may play an important role in functional recovery following burn injury. It is important to understand the time course and functional relevance of these brain changes following injury to optimize timely rehabilitation interventions and enhance functional recovery.

The aims of the current study were to investigate: (i) motor performance; (ii) neuroplasticity induced by paired associative stimulation (PAS); and (iii) intracortical inhibition within the motor cortex of individuals recovering during the subacute phase of burn injury (~3–12 weeks post-injury). We hypothesized that pegboard performance would be poorest at ~3 weeks post-injury and improve at ~6 to ~12 weeks post-injury. We also hypothesized that baseline intracortical inhibition and PAS-induced neuroplasticity would be altered in the burns group at ~3 weeks and that these differences would persist across the experimental protocol.

## Methods

### Participants

Thirty-four subacute burn patients were recruited consecutively through the Western Australian Adult Burn Unit databases at Fiona Stanley Hospital in Perth, Australia between January 2022 and April 2025. All patients meeting eligibility criteria during the recruitment period were invited to participate. While recovery of burn injury is considered a spectrum, for this study the subacute period was defined as 3–12 weeks following injury as it represents the time between the acute period (prior to complete epithelialization of the wound) and the chronic period (≥3 months following burn injury) [42]. An *a priori* power calculation was not conducted due to limited prior data to inform expected effect sizes for PAS-induced TMS outcomes in a subacute burn cohort; subgroup analyses (e.g. upper-limb burn injury cohort) were not powered for definitive inference and are presented as secondary analyses. Inclusion criteria included adult participants (age 18 years or older) with a non-severe burn injury defined as TBSA injury of <20% [43], no previous burn injuries, MEP elicited by single-pulse TMS, and flame, scald, contact, friction, chemical, or electrical burn. An additional inclusion criterion of a burn injury sustained no >8 weeks prior to enrolment in the study was applied to ensure that all participants would complete at least two experimental sessions. After participants provided their informed consent, they completed a 17-item TMS safety questionnaire, which was used to screen and exclude individuals with contraindications to TMS [44–46]. The study followed the Declaration of Helsinki guidelines and received ethical approval from the Murdoch University Human Research Ethics Committee (2021/047) and the South Metropolitan Health Service (RGS0000004279).

### Experimental protocol

Figure 1 shows the experimental protocol. The primary analyses in this manuscript focus on longitudinal change within the burn cohort; secondary comparisons with non-injured controls are reported in the Supplementary Materials (including control recruitment and assessment procedures). Participants who passed screening completed testing at three timepoints: (i) >5 weeks post-injury, (2) ~6 weeks post-injury, and (3) ~12 weeks post-injury. Assessment timing was necessarily opportunistic in this clinical cohort, resulting in an unbalanced dataset and relatively sparse coverage at the extremes of the observation window. Model-based margins were taken from the full model, and contrasts were evaluated at 23, 44, and 82 days (~3, 6, and 12 weeks) to align with clinical milestones and the densest sampling; these are analysis anchors, not fixed session time points. Each session lasted ~2.5 h and included neurophysiological testing and motor and sensory function assessments. Burn-specific and health-related well-being surveys were distributed electronically and completed within 24 h on either side of each session.

**Figure 1:**
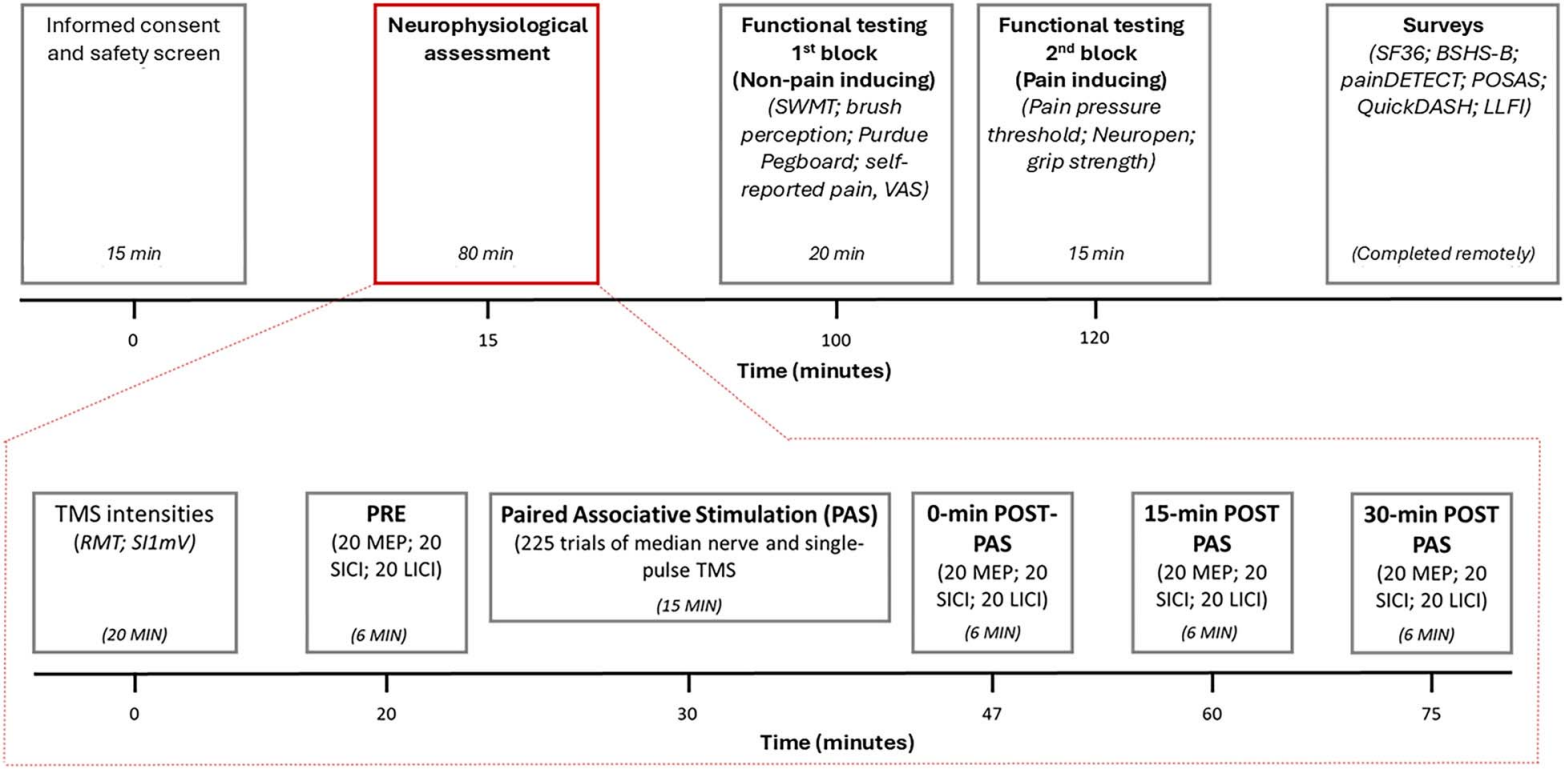
Timeline of the experimental protocol. *SWMT* Semmes–Weinstein monofilament test, *VAS* visual analog scale, *SF-36* short form-36, *BSHS-B* burn specific health scale-brief, *LLFI* lower limb functional index, *SI*_*1mv*_ stimulus intensity required to elicit MEPs with a mean peak-to-peak amplitude of ~1 mV. This figure is reproduced from earlier work with permission [33]

### Neurophysiological testing

#### Electromyography

Neurophysiological testing was conducted with participants seated comfortably with their arms supported by a pillow. EMG signals were predominantly recorded from the first dorsal interosseous (FDI) using Ag-AgCl surface electrodes in a belly-tendon configuration [47]. However, due to burn location over the FDI, one participant was recorded over the abductor digiti minimi (ADM), and another over the extensor digitorum muscle of the forearm [47]. The EMG signals were amplified (x1000) using a CED 1902, bandpass filtered (20–1000 Hz), and digitized at 5 kHz with a CED 1401 (Cambridge Electronic Design, UK). EMG data were collected from the hand (or arm) on the burn-affected side. If both sides were affected by the burn, the side with greater burn surface area and depth was identified as the burn-affected side for the purpose of testing. Hand dominance was determined by asking participants which hand they use for writing. Eighteen participants were tested on their left dominant hemisphere (right-handed), 13 on their right non-dominant hemisphere (right-handed), 2 on their right dominant hemisphere (left-handed), and 1 on their left non-dominant hemisphere (left-handed).

#### Transcranial magnetic stimulation

TMS was delivered using two MagStim 200^2^ stimulators connected via a MagStim BiStim module (MagStim, UK) to generate single and paired pulses. Monophasic pulses were administered through a 90-mm figure-of-eight coil positioned tangentially to the scalp. The coil handle was angled backward and rotated ~45° from the midline to induce a posterior–anterior current in M1, targeting the hemisphere contralateral to the most-affected hand. The optimal M1 stimulation site was determined as the location that consistently produced the largest MEPs in the target muscle in response to 10 suprathreshold single TMS pulses [46, 48]. This site was marked on a tight-fitting skull cap that was taped in a secure position to ensure consistent coil placement throughout testing. The resting motor threshold (RMT) was defined as the lowest stimulus intensity, expressed as a percentage of the maximum stimulator output (MSO), that elicited MEPs ≥50 μV in at least three of five consecutive trials [46]. SI_1mV_ was defined as the stimulus intensity needed to produce MEPs with a mean peak-to-peak amplitude of ~1 mV from 10 single TMS pulses. All measurements were taken with the target muscle at rest. The hotspot, RMT, and SI_1mV_ were determined in each testing session.

#### Intracortical inhibition

SICI and LICI were evaluated using single- and paired-pulse TMS protocols. For single-pulse trials, a TS was delivered at SI_1mV_. For SICI trials, a CS at 80% RMT preceded the TS at SI_1mV_ by a 3 ms inter-stimulus interval (ISI) [28]. For LICI trials, the CS and TS were both delivered at SI_1mV_, separated by a 100 ms ISI [49]. Trial conditions (SI_1mV_-alone, SICI, and LICI) were pseudo-randomized using Signal software (CED, UK), with a 5-second inter-trial interval (±20%). Each block consisted of 60 trials: 20 SI_1mV_-alone, 20 SICI, and 20 LICI. Each block lasted ~6 min. Participants were asked to stay quiet, keep their eyes open, and remain alert throughout the TMS testing. MEP amplitude, SICI, and LICI were measured with the target muscle at rest.

#### Paired associative stimulation

PAS was chosen to examine LTP-like neuroplasticity through spike-timing-dependent mechanisms [50]. PAS was administrated by pairing electrical stimulation of the median nerve on the most-affected side with single-pulse TMS applied to the contralateral M1 [51]. Peripheral nerve stimulation was delivered using 200 μs square-wave pulses via a direct current stimulator (Digitimer DS7, UK) and bipolar surface electrodes: the anode was positioned over the median nerve at the wrist, and the cathode 3 cm proximally. For three participants with wrist burns, the peripheral stimulating electrodes were placed on the palmar surface of the third digit on the most affected side, rather than over the median nerve at the wrist. The anode was positioned over the tip of the palmar surface of the third digit and the cathode 3 cm proximally. This electrode placement stimulated the median nerve via the palmar digital branches [52]. To determine sensory perceptual threshold, participants were instructed to report the first clear perception of the stimulus and a stepwise staircase procedure with two reversals was followed. Stimulation intensity was increased from a subthreshold intensity until perception was reported, then decreased until perception was lost (first reversal). This process was repeated to obtain a second reversal. The sensory perceptual threshold was calculated as the mean of the stimulation intensities at the two reversal points. The intensity of peripheral nerve stimulation for PAS was set to three times the sensory perceptual threshold. Single TMS pulses were applied to the contralateral M1 using a single MagStim 200^2^ stimulator at SI_1mV_, 25 ms after nerve stimulation. The PAS protocol consisted of 225 paired trials delivered at a frequency of 0.25 Hz over 15 min [50]. To direct participant attention during PAS, a light-emitting diode (LED) was placed 3–5 cm in front of the third digit of the target hand and flashed randomly in 20–40 trials throughout the protocol, as controlled by Signal software. Participants were instructed to focus on their hand during PAS and report the number of LED flashes after PAS finished [53].

Single-pulse MEP amplitude, SICI, and LICI were measured at four time points: before PAS (PRE), immediately after PAS (0 min POST-PAS), 15 min post-PAS (15 min POST-PAS), and 30 min post-PAS (30 min POST-PAS) ( Figure 1). Participants also rated their self-perceived alertness/sleepiness using the 10-step Karolinska Sleepiness scale after each testing block, including immediately after PAS [54]. To examine whether PAS-induced changes in LICI were associated with changes in sleepiness scores, we assessed the association between the PRE to 0 min POST-PAS change in the LICI ratio and the PRE to immediately post-PAS change in sleepiness score using a Spearman rank correlation on the full dataset. To account for within-participant dependence arising from repeated observations across sessions, confidence intervals (CIs) and *P*-values were derived using participant-level (cluster) bootstrapping (10 000 resamples), resampling participant IDs with replacement and retaining each participant’s full set of observations in every resample.

### Motor function: Purdue Pegboard

Motor performance was assessed using the Purdue Pegboard (Lafayette Instrument, USA), which consists of four subtests: unilateral dominant hand, unilateral non-dominant hand, simple bilateral, and bilateral assembly [55]. In the unilateral subtests, participants picked up and placed small pegs into vertically arranged holes on the pegboard in front of them, with the number of pegs placed within 30 seconds recorded for each hand. The testing order for the unilateral subtests was randomized. In the simple bilateral subtest, participants used both hands simultaneously to place pegs in pairs of holes, with the total number of completed pairs scored within 30 seconds. For the assembly subtest, participants alternated hands to assemble 4-item units (peg, washer, sleeve, washer) into the pegboard holes. The number of items in the completed assemblies within 60 seconds was recorded. Unilateral subtests were defined as dominant and non-dominant (rather than most-affected and least-affected) to avoid subgrouping complexity and loss of power, given the strong influence of hand dominance on outcomes [56]. Accordingly, bilateral pegboard performance was emphasized in the main manuscript to ensure that deficits on the most-affected side were reflected in the overall outcome. Participants were instructed to perform each task as quickly as possible.

### Surveys

All participants were asked to complete the surveys remotely via an email link (REDCap Electronic Database, USA) and completed within 24 h either side of each session.

#### SF-36

##### SF-36 health survey

Health-related quality of life was assessed using the SF-36 health survey, which is a validated self-report measure of physical and mental health [57–59]. The SF-36 comprises 36 items that assess health across eight domains. Items were scored and transformed according to standard SF-36 scoring procedures, with domain scores ranging from 0 to 100, where higher scores indicate better health status. In the current study, we calculated eight domain scores [57–59]: physical functioning (assesses the ability to perform daily physical activities, such as walking, climbing stairs, and lifting objects), role limitations due to physical health (measures the extent to which physical health problems interfere with work or other daily activities), role limitations due to emotional problems (measures the extent to which emotional problems affect work or other daily activities), energy/fatigue (captures energy levels and feelings of fatigue), emotional well-being (evaluates psychological well-being, including symptoms of depression, anxiety, and overall mood), social functioning (assesses the impact of physical and emotional health on social activities and interactions), pain (evaluates the intensity of pain and its impact on daily functioning), and general health (reflects overall self-perception of health, including current health status and future health expectations). These eight domain scores were used to calculate the Physical and Mental Component Summary scores, as described in the Data Processing section.

The SF-36 assessment is reported in the main manuscript; the Burn-specific Health Scale-Brief (BSHS-B), Quick Disabilities of the Arm, Shoulder, and Hand (QuickDASH), Lower Limb Functional Index (LLFI), Patient and Observer Scar Assessment Scale (POSAS), and painDETECT assessments are reported in the Supplementary Materials (Sections S4.1–S4.5).

### Perceptual pain, sensory function, and grip strength analysis

Testing of motor and sensory function was conducted in two blocks. Tests that were unlikely to generate a painful response were assessed in the first block (visual analog pain scale, Semmes-Weinstein Monofilament Test, brush touch perception, and Purdue Pegboard; administered in random order). Tests that might generate a painful response were assessed in the second block (Pain Pressure Threshold, Neuropen, and grip strength; administered in random order). Pegboard performance results are presented in the main manuscript, while the remaining functional assessments are detailed in the Supplementary Materials (Sections S4.6–S4.11).

### Data processing and statistical analysis

All single- and paired-pulse TMS trials were recorded using Signal software (CED, UK) for data acquisition and analysis. Peak-to-peak MEP amplitude was calculated from 10 to 60 ms following the stimulus (and 110–160 ms for LICI). The root mean square (RMS) of background EMG activity was assessed 100 ms preceding the stimulus. Trials were excluded from analysis if the background EMG activity exceeded the RMS mean plus two standard deviations (RMS + 2SD) of all trials in the session [60]. The median trial removal rate was 2.9%, with an interquartile range (25th–75th percentile) of 1.3% to 4.9% across all sessions.

For self-reported health and well-being, domain scores from the SF-36 were standardized using means and standard deviations from normative population data for gender and age [58, 61]. Z-scores for each of the eight subscales were calculated and weighted according to published factor scoring coefficients to derive the Physical Component Summary and Mental Component Summary scores. Composite scores were then transformed to have a mean of 50 and a standard deviation of 10, consistent with conventional scoring methods.

An a priori power calculation was not conducted because there are no data on the primary outcome measure in a sub-acute burn patient cohort to inform expected effect sizes for PAS-induced TMS outcomes. Statistical analyses were conducted in R (version 4.4.3) [62]. Primary data and code are available at OSF (view-only link: https://osf.io/n3euw/overview?view_only=a67c490f40864baea2dfce1ba1426490)*.*

#### Transcranial magnetic stimulation stimulator intensities

To examine whether TMS intensities changed over time, SI_1mV_ and RMT were modeled using linear mixed-effects models with a natural cubic spline for days since injury (DAYS) (degrees of freedom = 3) and participant-level random intercepts [63]. Spline models were preferred over linear models due to lower Akaike Information Criterion (AIC) values on comparison. Models with 3 degrees of freedom were selected to for estimation of non-linear trajectories across time. Normality, homoscedasticity, and linearity were assessed using residual plots and the performance diagnostic package [64] and all indicated adequate model fit. Marginal means (intensities) and trends (slopes) were computed at 23, 44, and 82 days using emmeans and emtrends [65], respectively (aligning with clinically meaningful milestones). Holm-adjusted comparisons were then used to evaluate time-dependent changes in stimulator intensity.

#### Modeling of intracortical inhibition and neuroplasticity over time

Analysis evaluated changes in intracortical inhibition over time. Generalized linear mixed models (GLMMs) were fitted using the lme4 package [63], specifying a Gamma distribution with a log link to model trial-level MEP amplitudes. The models included fixed effects for days since injury (DAYS, modeled using a natural cubic spline with 3 degrees of freedom), stimulation condition (CONDITION: single-pulse, SICI, LICI), and PAS block (BLOCK: PRE, 0, 15, and 30 min POST-PAS), including all interactions. Participant-specific intercepts and slopes for DAYS and CONDITION were included as random effects. Despite a minor convergence warning (max|grad| = 0.0176), results were consistent across optimizers, and the bobyqa-based model was retained [63]. Model fit was evaluated using the AIC and likelihood ratio tests. Residual plots stratified by CONDITION revealed mild heteroscedasticity and right-skew, particularly for single-pulse and LICI trials. However, no substantial violations of linearity or distributional assumptions were observed, and the residual patterns were consistent with the Gamma distribution specified in the model. Estimated marginal means (amplitudes) and marginal trends (slopes) were extracted at 23, 44, and 82 days post-injury using the emmeans and emtrends packages [65]. Instantaneous slopes, representing the first derivative (tangent) of the fitted curve, were used to assess the rate of change in MEP amplitude over time. Model-based contrasts estimated differences in marginal means and trends between SICI and LICI versus single-pulse trials, expressed on the response scale as ratios (marginal-mean ratios and slope ratios) interpretable as relative inhibition (<1) or facilitation (>1). Post hoc contrasts were performed within the PRE block and across PAS blocks with Holm correction. Percent changes for contrasts were also reported.

To remove the potential confound of PAS protocol variations in some participants (*n* = 3 with peripheral stimulation to the third digit), analyses were repeated excluding these participants, with analysis focused on PAS-induced intracortical inhibition measures.

#### Analysis of pegboard performance over time

To model changes in pegboard performance over time, separate linear mixed-effects models were fit for each subtest (dominant, non-dominant, simple bilateral, assembly), with DAYS modeled as a natural cubic spline (degrees of freedom = 3) and random intercepts for participants [63]*.* Spline models had low AIC values across all subtests and residual diagnostics confirmed appropriate model fits [64]*.* Both estimated marginal means (scores) and marginal trends (slopes) were extracted at 23, 44, and 82 days post-injury to capture changes in performance levels and the rate of change over time [65]. Slopes derived from the first derivative of the fitted spline were used to quantify the instantaneous rate of performance change across time. Holm-adjusted pairwise comparisons were used to evaluate differences across timepoints for both approaches. For the score-based analyses, percent change was calculated by dividing the estimated difference in marginal means by the earlier timepoint value, multiplied by 100. For the slope-based analyses, differences were based on estimated marginal trends extracted at 23, 44, and 82 days.

#### Changes in SF-36 physical and mental component summary scores over time

The Physical Component Summary and Mental Component Summary scores were calculated by adjusting SF-36 subscale scores against age and gender-based normative means and standard deviations from healthy individuals [58, 61]. To assess changes over time since injury, separate linear mixed-effects models incorporating natural cubic splines (degrees of freedom = 3) for days post-injury were fitted for Physical and Mental Component Summary scores using the lme4 package [63], including participant-specific random intercepts. Spline models had low AIC values for both outcomes. Residual diagnostics confirmed appropriate model fits [64], with no substantial violations of linearity, normality, or homoscedasticity. Mild right-skew and heteroscedasticity were observed for Mental Component Summary scores, but the deviations were within acceptable bounds for linear mixed-effects modeling [66]. Estimated marginal means (scores) and marginal trends (slopes) at Days 23, 44, and 82 were obtained using emmeans and emtrends [65], and pairwise comparisons between these timepoints were adjusted for multiple testing using the Holm method. For the score-based analyses, percent change was calculated by dividing the estimated difference by the earlier timepoint value and multiplying by 100. For the slope-based analyses, differences in the estimated daily rate of change were calculated between timepoints using model-derived estimates.

#### Time-varying associations between pegboard performance and baseline and neuroplastic transcranial magnetic stimulation outcomes and SF36 summary scores

To assess whether baseline intracortical inhibition could predict pegboard performance over time, separate models were fit for aggregated SICI and LICI amplitude ratios, each including an interaction between DAYS and simple bilateral or assembly pegboard subtests [63]. Assumptions of linearity and homoscedasticity were met for all four models [64]. While residuals deviated from normality, this is not considered a major concern, as linear mixed-effects models are generally robust to such violations [66]. No influential outliers were detected. Slopes of inhibition predicted by pegboard scores were extracted using the emtrends package at 23, 44, and 82 days [65], with day-specific significance determined when the 95% CI did not cross zero, and between-day comparisons assessed using Tukey-adjusted *P*-values. The same analytical approach was used to examine whether changes in single-pulse MEP amplitude following PAS (calculated as the difference between pre- and post-stimulation blocks, with all post-stimulation trials collapsed into a single block) could predict bilateral pegboard performance over time. Assumption checks confirmed that both the simple bilateral and assembly models demonstrated acceptable linearity and homoscedasticity, with residuals approximately normally distributed and no influential outliers identified [64].

An exploratory analysis was conducted to examine whether the association between PAS-induced changes in LICI and pegboard performance varied across the recovery period. PAS-induced change was calculated as the difference between baseline (PRE) and 30 min POST-PAS blocks. Separate linear mixed-effects models were fitted for the simple bilateral and assembly subtests, including an interaction between pegboard score and days since injury, with participant-level random intercepts [63]. Visual inspection of diagnostic plots indicated approximate linearity and no influential outliers for both models [64]. Mild heteroscedasticity and deviations from normality were observed; however, given the exploratory nature of the analysis and the robustness of linear mixed-effects models to such violations [66], these issues were not considered likely to meaningfully compromise model interpretation. Estimated marginal trends were extracted at 23, 44, and 82 days post-injury to assess the time-varying association between pegboard performance and LICI response [65]. Pairwise comparisons of these slopes were used to evaluate changes in the strength of association across time.

For all analyses, statistical significance was defined as *P* < 0.05. Data preprocessing and visualization were performed using R packages: dplyr [67], tidyverse [68], ggplot2 [69], and patchwork [70].

## Results

Data were collected at three timepoints: the first timepoint occurred a median of 19 days post-injury (range: 4–32 days); the second, 44 days (25–66 days); and the third, 86 days (69–124 days).

**Table 1:** Demographic and clinical characteristics of the subacute burn patients

**Age**	**Sex**	**Burn type**	**TBSA (%)**	**Burn depth**	**Burn location**	**Most affected side**	**Handedness**	**Sessions**		
								**1**	**2**	**3**
35	Male	Flame	2.00	Partial thickness	Hand, Neck	Right	Right	√	√	√
39	Male	Flame	1.75	Partial thickness	Hand, Upper arm	Right	Right	√	√	√
55	Male	Scald	1.00	Partial thickness	Hand	Right	Left	√	√	√
35	Male	Flame	0.30	Partial thickness	Hand	Right	Right	√	√	√
65	Female	Scald	0.10	Partial thickness	Hand	Right	Right	√	√	√
54	Female	Contact	0.09	Superficial	Hand	Left	Right	√	√	√
40	Female	Contact	0.01	Superficial	Hand	Right	Right	√	√	√
43	Male	Flame	11.00	Partial thickness	Forearm, Thigh, Lower leg, Foot	Left	Right	√	√	√
63	Female	Scald	2.50	Deep	Forearm, Upper arm	Right	Right	√	√	√
40	Female	Scald	1.00	Superficial	Forearm	Right	Right	√	√	√
24	Male	Scald	0.20	Partial thickness	Forearm	Left	Right	√	√	√
62	Male	Friction	0.10	Partial thickness	Forearm	Right	Right	√	√	√
30	Female	Scald	0.80	Superficial	Upper arm, Face	Right	Right	√	√	√
47	Female	Scald	3.00	Deep	Abdomen, Thigh	Right	Right	√	√	√
36	Male	Chemical	1.31	Deep	Abdomen, Thigh	Right	Right	√	√	√
63	Male	Chemical	1.25	Deep	Lower leg, Foot	Left	Right	√	√	√
33	Male	Scald	0.90	Deep	Lower leg	Left	Right	√	√	√
45	Female	Chemical	0.70	Deep	Abdomen, Thigh	Left	Right	√	√	√
44	Female	Contact	0.05	Partial thickness	Abdomen	Right	Right	√	√	√
39	Female	Scald	0.05	Superficial	Foot	Left	Right	√	√	√
59	Female	Scald	0.05	Partial thickness	Thigh	Left	Left	√	√	√
57	Female	Scald	0.01	Superficial	Face	Right	Right	√	√	√
46	Male	Scald	0.10	Superficial	Hand, Forearm	Left	Right	√	√	
62	Male	Contact	0.01	Superficial	Hand	Left	Left	√	√	
29	Male	Flame	0.20	Partial thickness	Lower leg	Left	Right	√	√	
29	Female	Contact	0.01	Deep	Face	Right	Right	√	√	
42	Male	Flame	0.22	Superficial	Hand, Forearm	Right	Right		√	√
56	Male	Scald	0.10	Partial thickness	Hand	Right	Right		√	√
18	Female	Contact	0.01	Superficial	Hand	Left	Right		√	√
36	Male	Flame	1.00	Deep	Forearm, Foot	Left	Right		√	√
31	Male	Contact	0.50	Superficial	Forearm	Right	Right		√	√
76	Male	Flame	3.00	Partial thickness	Lower leg	Left	Right		√	√
68	Female	Flame	1.00	Partial thickness	Face, Neck	Right	Right		√	√
64	Female	Scald	0.80	Superficial	Thigh	Left	Right		√	√

*TBSA* total body surface area

### Characteristics of subacute burn patients

Thirty-four subacute burn patients (16 females; age: 46.0 ± 14.4 years) participated in this longitudinal study. Twenty-two participants completed all three sessions; four completed <5 weeks and ~ 6 weeks; and eight completed ~6 and ~ 12 weeks. Days since injury was treated as a continuous variable across the observed range (4–124 days). Table 1 shows demographic and clinical characteristics of the burn participants. The median TBSA affected was 0.4 (range: 0.01–11.00; interquartile range: 0.91). Burn causes included contact (*n =* 7), scald (*n =* 14), flame (*n =* 9), chemical (*n =* 3), and friction (*n =* 1). Burn depths were classified as superficial (*n =* 12), partial-thickness (*n =* 14), deep (*n =* 8), and full-thickness (*n =* 0). Approximately two-thirds of injuries (*n =* 20) involved the upper limb—hand, forearm, or upper arm.

### Pegboard performance

#### Score differences

Pegboard performance was modeled over the observed range (4–124 days), with margins estimated and contrasts evaluated at 23, 44, and 82 days post-injury for each subtest. Figure 2 (top panel) shows estimated trajectories of bilateral pegboard performance during recovery. For the assembly subtest (B), performance improved significantly from Day 23 to 44 (+5.0%, adjusted *P* = 0.034), but no further changes were observed between Day 44 and 82 (−0.8%, adjusted *P* = 0.782), and the overall gain from Day 23 to 82 was not significant (+4.2%, adjusted *P* = 0.328). In contrast, simple bilateral scores (A) showed no significant improvement from Day 23 to 44 (+1.6%, adjusted *P* = 0.319) but declined significantly from Day 44 to 82 (−6.6%, adjusted *P* = 0.019).

**Figure 2:**
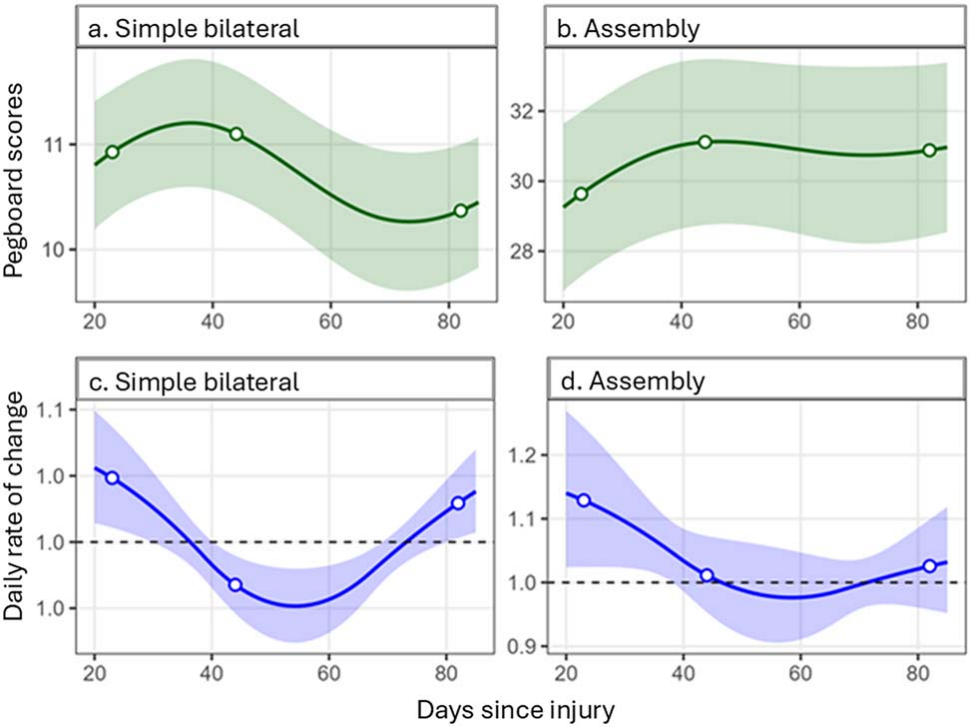
Panels **a** and **b** show modeled trajectories of score-level differences in pegboard task performance (a: simple bilateral; b: assembly) over time following injury. Shaded bands in (a) and (b) indicate 95% CIs. Panels **c** and **d** show the daily rate of change in pegboard task performance (c: simple bilateral; d: assembly) following injury. Curves show performance improvements or slowing over time: a value of 1.0 (dashed line) indicates no change; values >1.0 reflect improvement in pegboard performance over time; values <1.0 reflect reduced performance over time. Shaded bands in (c) and (d) represent uncertainty in the estimates. Open circles represent the estimates at 23, 44, and 82 days post-injury

For the dominant hand, scores improved significantly over time, with a + 4.9% increase from Day 23 to 44 (adjusted *P* = 0.012) and a + 8.7% increase from Day 23 to 82 (adjusted *P* = 0.006). There was no significant change between Days 44 and 82 (+3.7%, adjusted *P* = 0.142). Similarly, non-dominant hand performance improved, showing a + 4.4% increase from Day 23 to 44 (adjusted *P* = 0.18) and a + 5.7% increase from Day 23 to 82 (adjusted *P* = 0.044), with no further change between Days 44 and 82 (+1.2%, adjusted *P* = 0.583). Unilateral performance is shown in Supplementary Materials (Section S1, Figure S1).

#### Rate of change differences

Pegboard slope analysis revealed task-specific differences in recovery trajectories over time. Figure 2 (bottom panel) shows the modeled day-by-day change in bilateral pegboard task performance following injury. No significant differences in the daily rate of change were detected for the assembly task (all adjusted *P* ≥ 0.278). The simple bilateral subtest’s daily improvement rate significantly declined from Days 23 to 44 (−0.06 units/day, adjusted *P* = 0.005), indicating slower gains, followed by a significant increase in the rate of improvement from Days 44 to 82 (+0.05 units/day, adjusted *P* = 0.017). These results suggest a transient slowdown in bilateral performance gains ~6 weeks post-injury, which resolved by 82 days post-injury.

For the unilateral hand subtests, there were no significant changes in the daily rate of improvement between any timepoints (all adjusted *P* ≥ 0.447), indicating relatively stable recovery trajectories. Unilateral performance is shown in Supplementary Figure S1.

### SF-36 summary scores across recovery period

#### Score differences

Figure 3 (top panel) shows modeled trajectories of SF-36 Physical Component Summary and Mental Component Summary scores across days since injury. Marginal estimates were extracted and contrasts evaluated at 23, 44, and 82 days post-injury from models fit over the observed range (4–124 days). For the Physical Component Summary, a significant 8.1% increase was observed from Day 23 to Day 44 (adjusted *P* < 0.001), which was unchanged at Day 82 with a similar 8.2% increase compared to Day 23 (adjusted *P* = 0.009). No significant change occurred between Days 44 and 82 (adjusted *P* = 0.963). For the Mental Component Summary, the 3.7% increase from Day 23 to Day 44 was not significant (adjusted *P* = 0.121), while the 6.7% increase from Day 23 to Day 82 approached significance (adjusted *P* = 0.070). No significant difference was observed between Days 44 and 82 (adjusted *P* = 0.283). These results suggest early improvements in physical health-related quality of life during the first 6 weeks post-injury, with Physical Component Summary scores stabilizing and Mental Component Summary scores continuing to trend upward by 12 weeks. Physical Component Summary values remained above the population norm of 50, suggesting better-than-average perceived physical health relative to healthy individuals, whereas Mental Component Summary values were more closely aligned with population norms.

**Figure 3:**
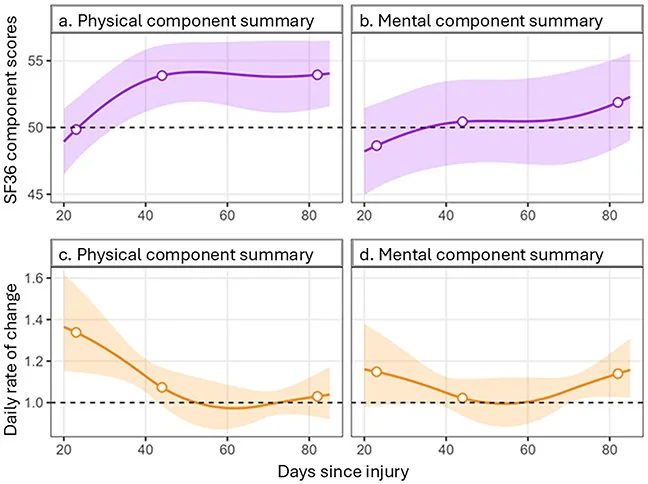
Panels **a** and **b** show estimated marginal means of SF36 physical and mental component summary scores over time post-injury. Scores are standardized to a population mean of 50 (SD = 10); the dashed line marks this reference. Panels **c** and **d** show estimated relative daily rates of change in physical and mental component summary scores over time. The dashed line at 1.0 indicates no change; values >1.0 reflect improvement, and values <1.0 indicate reduced recovery. For all panels, shaded bands represent 95% CIs. Open circles show the model-predicted values at Days 23, 44, and 82

**Figure 4:**
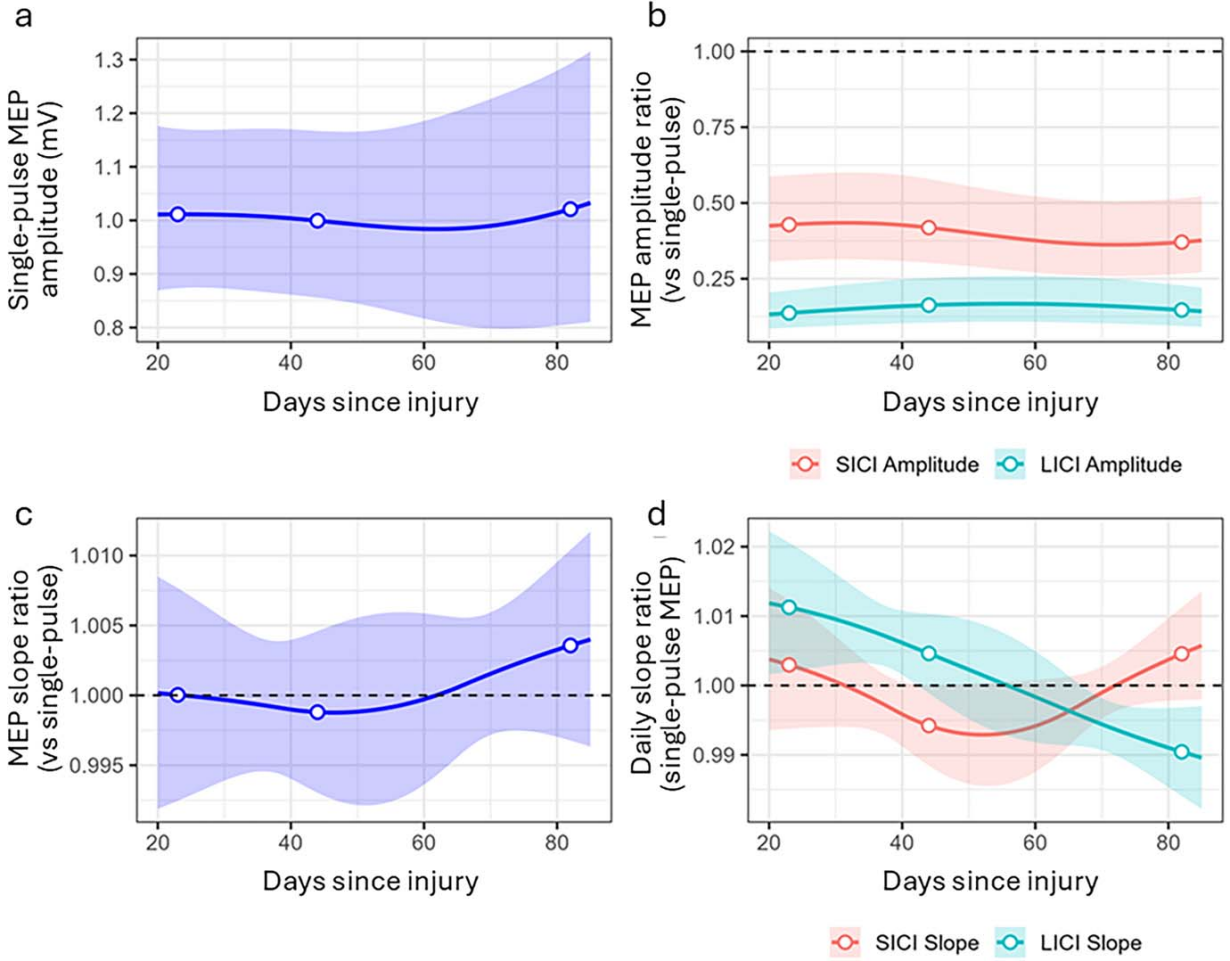
Panels **a** and **b** show modeled changes in single-pulse MEP amplitude (a) and SICI and LICI amplitude ratios (b) across days since injury during the baseline (PRE) block. SICI and LICI amplitude ratios (relative to single-pulse) <1 indicate inhibition compared to single-pulse trials, with smaller ratios indicating greater inhibition. Panels **c** and **d** show modeled changes in daily MEP slope ratios (a) and SICI and LICI slope ratios (b) across days since injury during the baseline (PRE) block. Values greater than 1.0 indicate an increase in single-pulse MEP amplitude over time, whereas values <1.0 reflect a decrease across days. For SICI and LICI slope ratios, values >1.0 indicate a relative reduction in inhibition over time, while values <1.0 reflect increasing inhibition across days. For all panels, shaded bands represent 95% CIs and open circles show modeled points correspond to key analysis days (23, 44, 82). *SICI* shor-intracortical inhibition, *LICI* long-intracortical inhibition

#### Rate of change differences

Figure 3 (bottom panel) shows instantaneous slopes in SF-36 Physical and Mental Component Summary scores at Days 23, 44, and 82 post-injury. For the Physical Component Summary, the rate of improvement declined from Day 23 to 44 (−0.22 units/day, adjusted *P* = 052.), approaching statistical significance, and was significantly lower by Day 82 compared to Day 23 (−0.26 units/day, adjusted *P* = 0.013). No significant difference was observed between Days 44 and 82 (−0.04 units/day, adjusted *P* = 0.641), indicating a plateau in physical recovery rate beyond ~6 weeks post-injury. For the Mental Component Summary, slope contrasts were not statistically significant across timepoints (e.g. 23 to 82: −0.01 units/day, adjusted *P* = 0.929), although the slope remained positive at Day 82.

## Neurophysiological measures

### Baseline transcranial magnetic stimulation intensities (resting motor threshold, SI_1mV_)

#### Baseline intensity differences

RMT and SI_1mV_ (stimulator output required to elicit a 1 mV MEP) were modeled as a function of days since injury. Margins were estimated from models fit over the observed range (4–124 days) and extracted—with contrasts evaluated—at 23, 44, and 82 days post-injury. SI_1mV_ remained stable over time, with percent changes ranging from −1.4% to +2.0%. No differences reached statistical significance after Holm correction (all adjusted *P* ≥ 0.080). RMT values showed similar stability, with no significant differences observed between any timepoints (all adjusted *P* = 1.000). Data are presented in Supplementary Materials (Section S2, Figure S2).

#### Rate of change differences

Pairwise contrasts of slope estimates showed no statistically significant changes in corticospinal excitability (SI_1mV_) across timepoints (adjusted *P* ≥ 0.102). No contrasts for RMT were significant (all adjusted *P* = 1.000), indicating no evidence of time-dependent changes in RMT across recovery. Data are presented in Supplementary Materials (Section S2, Figure S2).

### Short- and long-intracortical inhibition in subacute burn patients

#### Baseline Intracortical inhibition changes

Figure 4 (top panel) show modeled trajectories of single-pulse MEP amplitude and intracortical inhibition (SICI and LICI) amplitude ratios over time during the baseline (PRE) block. We evaluated marginal mean MEP amplitudes, SICI, and LICI ratios during the baseline (PRE) block at 23, 44, and 82 days post-injury. At all three time points, both SICI and LICI conditions showed inhibition relative to single-pulse trials. Single-pulse trial amplitude estimates were close to 1.0 at each time point: pairwise comparisons indicated no significant differences across time (all adjusted *P* = 1.000). Single-pulse MEP amplitude remained stable across the subacute period and provided a stable reference for evaluating SICI and LICI.

Both SICI and LICI amplitude ratios remained stable across time. Pairwise comparisons showed no significant differences in amplitude for either SICI or LICI ratios across days (all adjusted *P* = 1.000).

**Figure 5:**
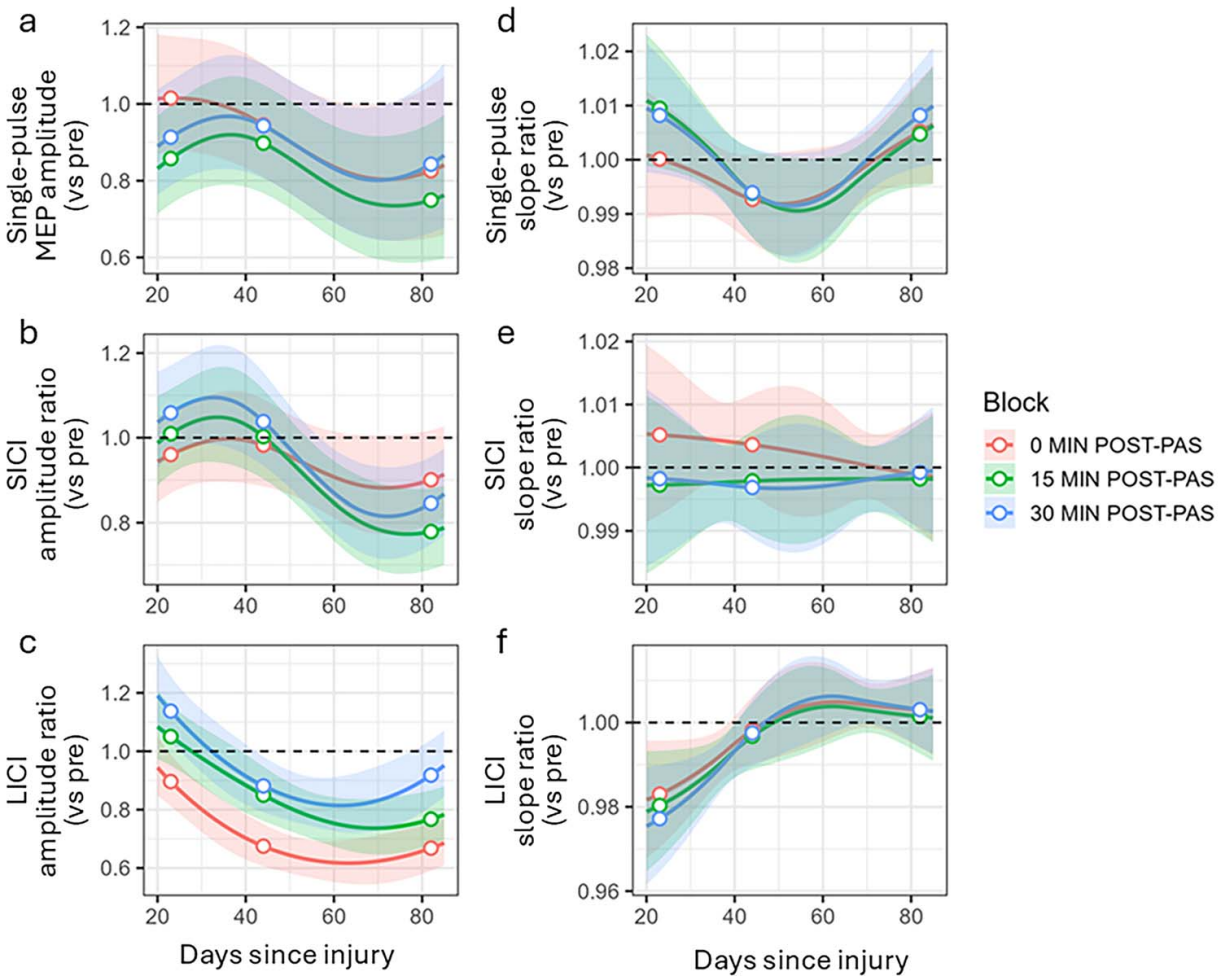
Left panel shows modeled changes in MEP amplitude (**a**), SICI (**b**), and LICI (**c**) following PAS, expressed relative to the PRE block (dashed line at ratio = 1.0). Ratios <1.0 reflect increased inhibition relative to the PRE block. Block conditions are color-coded: 0 min POST-PAS (red), 15 min POST-PAS (green), and 30 min POST-PAS (blue). Right panel shows model-estimated slope ratios (relative to the PRE block) for single-pulse (**d**), SICI (**e**), and LICI (**f**) conditions following PAS at 0, 15, and 30 min POST-PAS. Ratios <1.0 indicate reduced daily MEP slope compared to PRE. For all panels, shaded bands represent 95% CIs, and open circles indicate values at key assessment days (23, 44, and 82 days post-injury). *SICI* shor-intracortical inhibition, *LICI* long-intracortical inhibition, *PAS* paired associative stimulation

#### Rate of change differences

Figure 4 (bottom panel) shows the day-by-day change in MEP slope ratios during the baseline (PRE) block following injury. PRE-block marginal slopes were estimated from models fit over the observed range (4–124 days) and evaluated at 23, 44, and 82 days post-injury. Slope estimates for single-pulse trials indicated stable corticospinal excitability across the subacute period. No significant differences were found between days (all adjusted *P* ≥ 0.887).

No significant changes were detected in the SICI slope ratio (relative to single-pulse trials); there was a modest increase from Days 44 to 82 (+0.010 units/day, adjusted *P* = 0.065) that did not reach significance. For LICI, slope ratios decreased significantly from Day 23 to 82 (−0.021 units/day, adjusted *P* < 0.001) and from Day 44 to 82 (−0.014 units/day, adjusted *P* = 0.002), indicating a progressive increase in LICI over the subacute period. Values >1.0 with a negative slope indicate that LICI is increasing over time (i.e. reduced inhibition is reversing), while values <1.0 with a negative slope reflect further increases in LICI, indicating progressively stronger inhibition.

### Single-pulse motor evoked potential amplitude and Intracortical inhibition following paired associative stimulation

To direct participant attention during PAS, a LED flashed randomly throughout the protocol; participants were instructed to focus on their hand during PAS and report the number of LED flashes after PAS finished. The mean absolute error between reported and true counts was 1.9, with a standard deviation of 2.8. No participants were excluded from the analysis due to errors in LED counting.

#### Amplitude differences

Figure 5 (left panel) shows the day-by-day changes in corticospinal excitability and intracortical inhibition following PAS, shown relative to the PRE block. Marginal mean MEP amplitudes were estimated from models fit over the observed range (4–124 days) and evaluated across post-PAS blocks (0, 15, and 30 min POST-PAS) at 23, 44, and 82 days post-injury. Single-pulse amplitudes showed small, non-significant changes across time (all adjusted *P* ≥ 0.916), indicating that corticospinal excitability following PAS remained stable over the subacute recovery period.

SICI amplitude ratios showed no significant changes across any post-PAS block or timepoint (all adjusted *P* ≥ 0.606). In contrast, LICI amplitude ratios showed a significant increase in inhibition from Day 23 to 44 at 15 min POST-PAS (−22.7%, adjusted *P* = 0.044) and 30 min POST-PAS (−24.9%, adjusted *P* = 0.020). No other LICI comparisons reached significance (all adjusted *P* ≥ 0.121), suggesting that the effect was transient.

#### Rate of change differences

Figure 5 (right panel) shows the day-by-day changes in corticospinal excitability and intracortical inhibition slope ratios following PAS, shown relative to the PRE block. We examined slope estimates for single-pulse trials, SICI, and LICI across the post-PAS time period. Single-pulse trial slope comparisons showed modest changes over time, with a trend toward reduced excitability from Day 23 to 44 at 15 min POST-PAS (−0.016 units/day, adjusted *P* = 0.077) and 30 min POST-PAS (−0.014 units/day, adjusted *P* = 0.079), followed by a partial return from Day 44 to 82 at 30 min POST-PAS (+0.014 units/day, adjusted *P* = 0.079). Overall, the rate of change in corticospinal excitability remained stable throughout the subacute period following PAS.

SICI slope contrasts were non-significant across all pairwise comparisons (all adjusted *P* = 1.000). LICI slope ratios progressively increased, consistent with a time-dependent reduction in the PAS-induced increase in LICI. Significant increases in LICI slope values from Day 23 to 82 were observed across all post-PAS blocks: at 0 min POST-PAS (+0.020 units/day, adjusted *P* = 0.036), 15 min POST-PAS (+0.021 units/day, adjusted *P* = 0.026), and 30 min POST-PAS (+0.026 units/day, adjusted *P* = 0.003). An additional significant increase was also observed from Day 23 to 44 at 30 min POST-PAS (+0.021 units/day, adjusted *P* = 0.016). Other comparisons did not reach significance following correction (e.g. 15 min POST-PAS: 23 to 44, +0.017 units/day, adjusted *P* = 0.069).

In summary, amplitude-ratio results show that PAS increased LICI within-session, with the largest increase around Day 44. However, rate of change (slope) results across days show that the magnitude of this PAS-related increase decreased from Day 23 to Day 82. Taken together, PAS-induced LICI peaks near Day 44 and then decreases over time.

Alertness can fluctuate across intensive testing sessions and may influence PAS responsiveness [53]; therefore, we examined whether changes in self-reported sleepiness were associated with PAS-induced changes in LICI using data from the full dataset. Results showed that the PAS-induced change in the LICI ratio from PRE to 0 min POST-PAS was negatively associated with the change in sleepiness scores from PRE to immediately post-PAS (Spearman *rho* = −0.27). Because participants contributed repeated observations, inference was based on participant-level (cluster) bootstrapping (10 000 resamples; resampling IDs and retaining all observations per participant). The 95% bootstrap CI was entirely below zero (−0.46 to −0.06) and the two-sided bootstrap *P*-value was 0.017, indicating that larger increases in sleepiness scores were associated with more negative (i.e. smaller) PRE-to-POST-PAS changes in the LICI ratio (see Supplementary Materials Figure S7).

### Sensitivity analysis: exclusion of non-standard testing configurations

As a sensitivity analysis, we repeated the analysis using a sensitivity model focused on PAS-induced intracortical inhibition outcomes, after excluding burn participants tested under non-standard procedures or measurement configurations (*n =* 3 with peripheral stimulation to the third digit).

#### Amplitude differences

LICI amplitude ratios showed no significant changes from Day 23 to 44 in any post-PAS block (all adjusted *P* ≥ 0.175). However, a delayed increase in inhibition was observed from Day 44 to 82 at 30 min POST-PAS, where LICI (relative to PRE) became more inhibitory at Day 82 compared with Day 44 (+48.7%, adjusted *P* = 0.011). No other LICI day-to-day comparisons reached significance (all adjusted *P* ≥ 0.175), and SICI showed no significant changes across days in any block (all adjusted *P* ≥ 0.279).

#### Rate of change differences

SICI slope contrasts were non-significant across all pairwise comparisons in the sensitivity model (all adjusted *P* ≥ 0.700). For LICI, the evidence was more limited and concentrated at 30 min POST-PAS: LICI slope values increased from Day 23 to Day 44 (+0.037 units/day, adjusted *P* < 0.001) and from Day 23 to Day 82 (+0.027 units/day, adjusted *P* = 0.006), consistent with a time-dependent reduction in the PAS-induced increase in LICI. All other LICI slope day comparisons did not reach significance after Holm correction (e.g. 0 min POST-PAS: adjusted *P* = 0.271; 15 min POST-PAS: adjusted *P* = 0.117), and no 44–82 LICI slope contrasts were significant (all adjusted *P* ≥ 0.203).

### Baseline short- and long-intracortical inhibition and bilateral pegboard performance

Figure 6 shows the estimated associations between baseline intracortical inhibition and bilateral pegboard subtest scores at each timepoint. For SICI amplitude ratios, the association with simple bilateral performance was positive at all timepoints, with the steepest slope observed at Day 23 [+0.089 units per unit increase, 95% CI (0.003, 0.175)]. Pairwise comparisons of these slopes across days did not reach significance after correction (all adjusted *P* ≥ 0.390). The association with SICI and assembly performance was weak and nonsignificant at all timepoints, with no significant between-day differences (all adjusted *P* ≥ 0.949). For LICI amplitude ratios, the association with both simple bilateral performance and assembly performance was weak and not statistically significant at any timepoint (all adjusted *P* ≥ 0.778).

**Figure 6:**
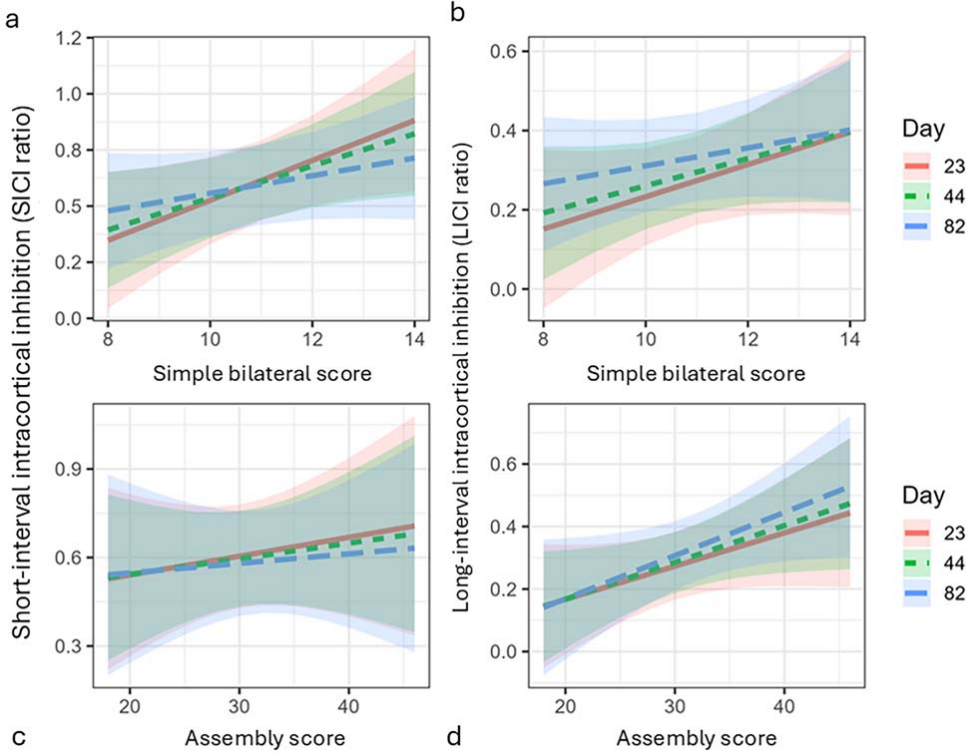
Estimated relationships between bilateral pegboard subtest scores and inhibition amplitude ratios across timepoints. (**a** and **b**) display relationships between simple bilateral scores and SICI (a) and LICI (b). (**c** and **d**) show relationships between assembly scores and SICI (c) and LICI (d). Lines represent model-estimated slopes, and shaded bands represent 95% CIs. *SICI* shor-intracortical inhibition, *LICI* long-intracortical inhibition

### Single-pulse motor evoked potential amplitude following paired associative stimulation and bilateral pegboard performance

Linear mixed models were used to examine whether bilateral pegboard task performance was associated with changes in single-pulse MEP amplitude following PAS. For both the simple bilateral subtest and the assembly subtest, no significant associations were observed between task performance and changes in MEP amplitude at any timepoint, and no differences in the strength of association were detected across days (all adjusted *P* ≥ 0.609). Together, these results suggest that changes in corticospinal excitability following PAS were not related to bilateral pegboard task performance at any of the assessed timepoints. Data are presented in Supplementary Materials (Section S3, Figure S3).

### Long-intracortical inhibition ratio change following paired associative stimulation and bilateral pegboard performance

Given the significant change in LICI following PAS, and the difference in PAS-induced LICI across days post-injury, day-specific slope estimates were derived to examine how changes in LICI following PAS (PRE—30 min POST-PAS) related to bilateral pegboard performance at 23, 44, and 82 days post-injury. Figure 7 shows model-estimated associations between changes in LICI following PAS and bilateral pegboard performance at each timepoint. For simple bilateral performance, a significant negative association was observed at Day 44, (95% CI: −0.073 to −0.004), indicating greater PAS-induced LICI increase was associated with better simple bilateral subtest performance. Non-significant associations were observed between simple bilateral subtest performance and PAS-induced change in LICI at 23 and 82 days post-injury (23 days: 95% CI: −0.089 to 0.001; 82 days: 95% CI: −0.064 to 0.007). Pairwise contrasts of slopes across days revealed no statistically significant differences (all adjusted *P* ≥ .802), suggesting that the strength of the relationship between LICI and sequenced bilateral performance was unchanged over the recovery period. For the assembly task, no significant associations were detected at any timepoint. Pairwise contrasts across days were likewise non-significant (all adjusted *P* ≥ 0.576), reflecting stable associations over time.

**Figure 7:**
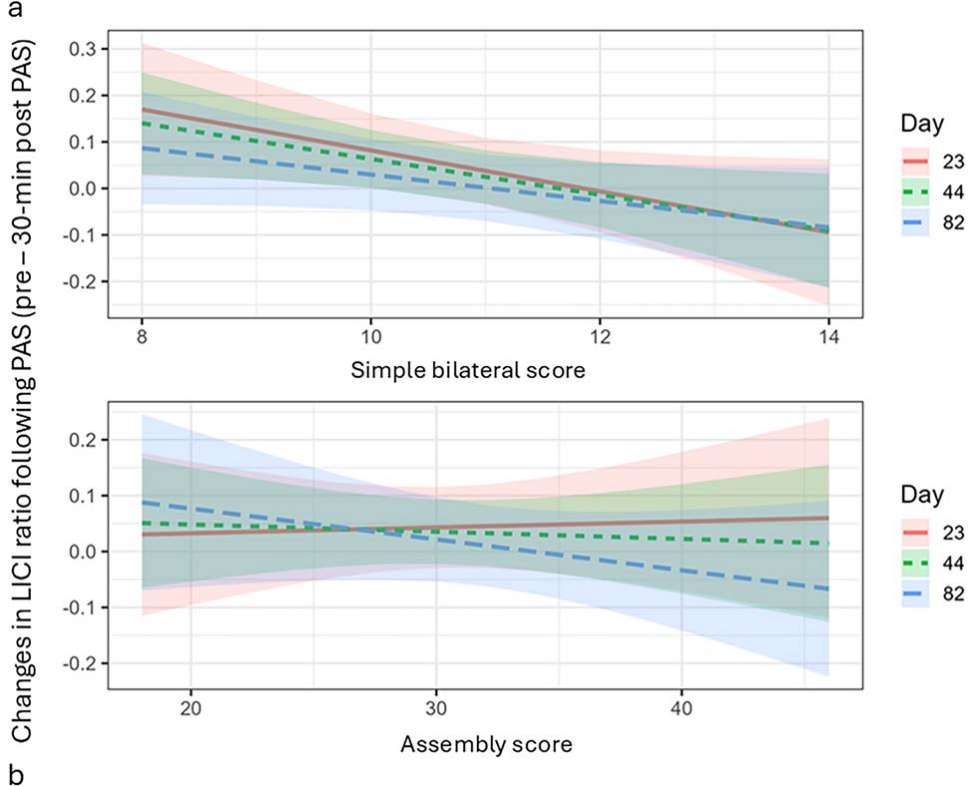
Model-estimated associations between changes in LICI amplitude ratio following PAS (PRE—30 min POST-PAS) and simple bilateral (**a**) and assembly (**b**) pegboard performance across Days 23, 44, and 82 post-injury. Shaded bands represent 95% CIs for each day-specific slope estimate. *LICI* long-intracortical inhibition, *PAS* paired associative stimulation

### Sleepiness scores following paired associative stimulation

Participants reported a significant increase in sleepiness following PAS. Compared to PRE, sleepiness was significantly higher at all post-PAS blocks (all adjusted *P* ≤ 0.049), peaking immediately after PAS and gradually declining over time, with sleepiness in the final post-PAS block significantly lower than immediately after PAS (adjusted *P* = 0.002).

## Discussion

This study examined motor performance, neuroplasticity, and motor cortex inhibition changes during the first 12 weeks following non-severe burn injury. There were three key results. First, improvements in motor performance and overall physical and mental functioning were greatest between ~3 and ~ 6 weeks following burn injury and then plateaued to ~12 weeks following burn injury. Second, baseline resting-state, LICI progressively increased across the first ~12 weeks following injury, suggesting increasing GABA_B_-mediated inhibition over the studied period. Third, PAS increased LICI at 30 min following stimulation and the magnitude of this PAS-induced increase in LICI gradually reduced over the first ~12 weeks following burn injury, suggesting PAS increased GABA_B_-mediated inhibition with the greatest change early following burn injury. In addition, the PAS-induced increase in LICI was associated with bilateral motor performance, such that a greater PAS-induced increase in LICI was associated with better bilateral motor performance.

### Subacute functional recovery trajectories after non-severe burn injury

Unilateral dominant and non-dominant hand performance and the complex bilateral assembly task performance increased from ~3 to ~6 weeks following injury and then plateaued to ~12 weeks following injury. The current findings are consistent with previous self-report data, measured by the QuickDASH, showing improvements in upper-limb motor function 1 to 3 months following non-severe burn injuries [3]. Together, these findings are consistent with data showing significant positive correlations between self-report and objective measures of hand function ~18 months following burn injury [71]. Our findings extend this by providing objective motor performance improvements in the subacute period. This improvement in motor performance is similar to previous findings in the subacute period for severe burn injuries [72], and consistent with previous research showing no difference in fine motor control, measured using the self-report Burn Outcome Questionnaire, between severe and non-severe burn injury patients 3 years following injury [73]. This may indicate that the early post-burn period presents an important window of opportunity for interventions aimed at optimizing the neuroplastic response; future research should examine associations between early changes in limb use and longer-term motor outcomes following burn injury.

A significant decline in simple bilateral performance was evident between ~6 and ~ 12 weeks. The reason for the decline in simple bilateral performance but not complex bilateral performance is unclear but could reflect difficulties in timing and synchronization between hands required for the synchronous bilateral task, which depend on interhemispheric connectivity [74, 75]. This, however, is speculative: it is important to characterize interhemispheric connectivity and associations with bilateral motor performance post-injury.

Physical scores increased significantly ~3 and ~ 6 weeks post-injury and then plateaued, while mental health scores gradually trended upward, suggesting that perceived health and well-being recover most rapidly in the early subacute phase following non-severe burn injury. This is consistent with meta-analytic findings showing the greatest improvements in health-related quality of life during the first ~6–8 weeks post-injury [76] and previous research showing that individuals with non-severe injuries report faster functional recovery and quicker return to daily activities than severe burn patients [76], and that health-related quality of life scores in individuals with non-severe burns often remain within normative ranges [1]. In supplementary between-group analyses, SF-36 scores at ~12 weeks post-injury showed no difference compared with non-injured controls (see Supplementary Materials Section S5.3 and Figure S5.3). The SF-36 Physical Component Summary scores were above the normative population mean of 50 [61, 77], indicating higher perceived health and well-being compared to the general population, which might reflect a self-selection bias: individuals participating in health research have high levels of education, employment, and health compared to individuals who do not participate in research [78].

### Increased baseline long-intracortical inhibition but not short-intracortical inhibition ~3 to ~12 weeks following burn injury

Baseline SICI amplitude ratios remained stable across time, suggesting no meaningful change in GABA_A_-mediate inhibition between ~3 and ~ 12 weeks following non-severe burn injury [25, 30, 79]. These findings replicate prior reports of no change in SICI from 6–12 weeks [34] and extend them by showing no difference at ~3 weeks in our cohort. In the current study, secondary between-group analyses indicated that SICI was higher in non-injured controls than burn patients at ~3 and ~ 6 weeks (Holm-adjusted *P* = .048 and .045) but showed no clear difference at ~12 weeks (see Supplementary Materials Section S5.4 and Figure S5.4). This might suggest a reduction in SICI up to ~6 weeks following burn injury, which is normalized by ~12 weeks following burn injury; however, this finding must be interpreted with caution as the burn samples across timepoints are semi-independent and only a single control timepoint was included in the analysis. In the current study, slope-based analysis revealed no change in SICI slope ratios over time (in the full sample and sub-sample of upper-limb burn patients: see Supplementary Materials Section S6.1). Previous research has shown no difference in SICI between burn patients 1–3 years following injury and controls [33]. Together, these findings suggest GABA_A_-mediated intracortical inhibition remains largely unchanged following burn injury.

Baseline LICI amplitude ratios remained stable across time, suggesting no change in GABA_B_-mediated inhibition between ~3 and ~ 12 weeks following non-severe burn injury [25]. Secondary between-group analyses indicated that LICI was higher in non-injured controls than burn patients at ~6 weeks (Holm-adjusted *P* = .039) but showed no clear difference at ~12 weeks (see Supplementary Materials Section S5.4 and Figure S5.4). This might suggest a reduction in LICI ~6 weeks following burn injury, which is normalized by ~12 weeks following burn injury; however, this finding must be interpreted with caution as the burn samples across timepoints are semi-independent and only a single control timepoint was included in the analysis. Previous research examining cSP duration, which reflects both GABA_A_- and GABA_B_-mediated inhibition [80], reported no significant differences between burn participants and non-injured controls [32]. It is worth noting, however, that Garside *et al.* [32] reported exploratory subgroup analyses (*n =* 13) showing shorter cSP durations on the burn-affected side in individuals with upper-limb burns, suggesting a reduction in long-acting inhibition following burn injury. In the current study, subgroup analysis on upper limb participants showed no change in LICI from ~3 to ~12 weeks following injury. These inconsistent findings could be due to differences in time since injury of the two studies: while the current study longitudinally measured LICI from ~3 to ~12 weeks following injury, the participants in Garside *et al.* [32] ranged from 6 weeks to 8 years following burn injury. It is also possible that there are differences in inhibition when measured during voluntary activation between burn patients and controls. Future research should examine task-related changes in intracortical inhibition in burn patients. The slope-based analysis in the current study revealed a significant reduction in LICI slope ratios over time, suggesting a time-dependent increase in GABA_B_-mediated inhibition from ~3 to ~12 weeks following non-severe burn injury. Taken together, these results suggest that while baseline intracortical inhibition remains stable at the output level, slope-based analyses may capture time-dependent changes in cortical inhibitory function during the subacute period.

### Greater baseline short-intracortical inhibition associated with poorer bilateral performance early in recovery

Individuals with greater SICI performed poorly on the simple bilateral subtest at ~3 weeks but not ~6 or ~ 12 weeks following injury. Although there was no change in SICI from ~3 to ~12 weeks following injury, the magnitude of SICI at an individual level is associated with bilateral performance. In burn patients 1–3 years following injury [33], and in non-injured individuals [81], greater SICI is associated with better performance on the Purdue pegboard. Taken together, these findings suggest that the functional role of GABA_A_-mediated inhibition differs across the subacute and chronic periods following a burn injury, with an atypical association of greater GABA_A_-mediated inhibition and poorer manual dexterity very early following a burn injury, which normalizes to the typical association of greater GABA_A_-mediated inhibition and better manual dexterity 1–3 years following a burn injury. Future research should examine whether early-recovery SICI and manual dexterity are predictive of long-term post-burn outcomes.

In this study, we found no evidence of an association between LICI and manual dexterity. Prior work shows that greater LICI 1–3 years post-injury is linked to better dexterity [33], suggesting GABA_B_-mediated inhibition may relate to motor performance in the chronic but not subacute period. These null results should be interpreted cautiously: the study was powered to detect differences, not equivalence, and the modest sample yields wide CIs, so small effects cannot be excluded. In addition, pegboard performance in some participants was constrained by hand injury and dressings. Associations between inhibition and dexterity might have been diluted by these injury-related performance constraints and, therefore, weak or null inhibition–dexterity associations in the subacute period should not be taken as evidence against a relationship. We found no evidence that SICI or LICI were associated with SF-36 Physical or Mental Component Summary scores from ~3 to ~12 weeks post-injury, indicating no detectable link between GABAergic inhibition and self-reported general function in the subacute period.

### Motor evoked potential unchanged and long-intracortical inhibition increased following paired associative stimulation

We focused on neuroplasticity driven by sensorimotor integration in individuals with burns, as sensory dysfunction can impair motor function and long-term outcomes [82, 83]. In the current study, PAS was chosen to assess LTP-like plasticity due to its role in sensory-motor integration [51], which may be particularly relevant following burn injury where sensory and motor dysfunction is common, and its effectiveness in increasing MEP amplitude [50]. Following PAS, single-pulse MEP amplitude and slope ratios remained stable, suggesting no systematic change in corticospinal excitability induced by PAS ~3 to ~12 weeks following burn injury. Therefore, the current findings suggest PAS-induced LTP-like plasticity is reduced or impaired in the subacute period following burn injury and extends previous work showing no evidence of PAS-induced LTP-like plasticity in burn patients 1–3 years following injury [33]. Previous research examining neuroplasticity in M1 in the subacute period following burn injury has shown reduced LTD-like plasticity induced by spaced cTBS at 6 weeks but not at 12 weeks post-injury [41], suggesting a reduced capacity for LTD-like neuroplasticity ~6 weeks following burn injury, which normalizes by ~12 weeks following burn injury. In secondary between-group analyses, non-injured controls showed a greater PAS-induced increase in MEP amplitude than burn patients at ~12 weeks post-injury (see Supplementary Materials Section S5.5 and Figure S5.5.1), which might suggest reduced PAS-induced LTP-like plasticity at ~12 weeks following burn injury. However, this finding must be interpreted with caution as the burn samples across timepoints are semi-independent and only a single control timepoint was included in the analysis. Furthermore, it is important to consider these findings of no PAS-induced change in corticospinal excitability in the context of inconsistent results from previous PAS studies in healthy individuals: some studies have shown increased MEP amplitude post-PAS (for example see: [51]) others have shown no change in MEP amplitude post-PAS (for example see: [84]), and there are reports of high response variability, with only ~50% of participants responding as expected [85].

In the current study, there was no PAS-induced change in SICI amplitude or slope ratios ~3 to ~12 weeks following burn injury, suggesting no modulation of GABA_A_-mediated inhibition by PAS. This is consistent with research in healthy individuals, showing no change in SICI following PAS [86–88] and previous work in burn patients 1–3 years following burn injury [33]. Taken together, this suggests that PAS does not modulate the excitability of GABA_A_-mediated inhibitory circuits in M1, and this is not affected in either subacute or chronic post-injury periods for burn patients.

Following PAS, LICI amplitude ratios showing increased at 15 and 30 min POST-PAS ~6 weeks following injury, indicative of enhanced GABA_B_-mediated inhibition [25]. This finding of increased LICI post-PAS is consistent with previous research showing a short-lasting increase in LICI post-PAS (e.g. at 0 and 15 min POST-PAS) in burn patients 1–3 years post-non-severe injury [33], and research in healthy young individuals that has shown increased LICI following PAS [89]. Secondary analyses indicated that non-injured controls showed an increase in LICI following PAS (see Supplementary Materials Section S5.5 and Figure S5.5.3). It is important to note that one previous study found no change in LICI following PAS in healthy adults when using stimulation parameters comparable to the current study [90], suggesting changes in LICI following PAS might show high inter-individual variability in responses, similar to the changes in MEP amplitude following PAS.

Slope-based metrics revealed a progressive reduction in the PAS-induced increase in LICI over time, with slopes becoming less negative from ~3 to ~12 weeks across all post-PAS blocks, indicating that PAS-induced modulation of GABA_B_-mediated inhibition varies within the first ~12 weeks after injury, with the PAS-related increase in LICI weakening over time. Although we did not directly measure inhibitory neurotransmission, LICI assessed with paired-pulse TMS is commonly interpreted as a putative index of GABA_B_ receptor–mediated intracortical inhibition, supported by converging pharmacological and physiological evidence [25, 49, 91]. The observed PAS-related changes in LICI likely reflect modulation of GABA_B_-mediated inhibitory circuit excitability. It is worth noting that the functional significance of the PAS-induced increase in GABA_B_-mediated inhibition remains uncertain; while PAS-induced LICI modulation may reflect adaptive inhibitory capacity within M1, further research is needed to determine how such neurophysiological effects relate to recovery of motor function following burn injury. In addition, future research using multimodal measures of GABAergic inhibition (e.g. magnetic resonance spectroscopy–derived GABA concentration estimates, TMS-EEG, or complementary paired-pulse TMS protocols) could better delineate the neurochemical and circuit mechanisms underpinning LICI modulation in this population.

To remove the potential confound of PAS protocol variations in some participants (*n =* 3 with peripheral stimulation to the third digit), we repeated the amplitude- and slope-based PAS-induced intracortical inhibition analyses using the sensitivity model. For amplitude–based inhibition ratios, the overall pattern was similar across the two models: SICI showed no Holm-adjusted significant day-to-day changes, and most LICI day comparisons were non-significant. In the full-sample model, LICI showed a significant increase in inhibition between Day 23 and Day 44 at 15 min and 30 min POST-PAS (−22.7%, adjusted *P* = .044; −24.9%, adjusted *P* = .020), with all other LICI day contrasts not reaching significance (all adjusted *P* ≥ .121). In the sensitivity model, these Day-23-to-Day-44 effects at 15- and 30-min POST-PAS were attenuated and not significant after Holm correction (adjusted *P* = .310 and .175), and the only Holm-significant LICI day contrast was observed between Day 44 and Day 82 at 30-min POST-PAS (+48.7%, adjusted *P* = .011). Taken together, these results suggest the amplitude-based PAS-related day-to-day LICI effects may be sensitive to testing procedures and measurement configuration and should be interpreted cautiously.

For slope–based inhibition ratios, the overall pattern was similar across the two models: all SICI slope day-to-day contrasts were non-significant after Holm correction (full-sample: all adjusted *P* = 1.000; sensitivity: all adjusted *P* ≥ .700), while LICI showed selective evidence of increasing slope contrasts over time, consistent with a time-dependent attenuation of the PAS-related LICI effect. In the full-sample model, LICI slope contrasts increase significantly between Day 23 and Day 82 across all post-PAS blocks, with an additional significant increase between Day 23 and Day 44 at 30 min POST-PAS. In the sensitivity model, the only Holm-significant changes were at 30 min POST-PAS, between Day 23 and Day 44. Taken together, both models show no clear day-to-day effects for SICI and that slope-based LICI changes are likely sensitive to sample and/or testing-procedure differences, and should be interpreted cautiously.

Greater increase in sleepiness scores from PRE to immediately post-PAS was associated with a more negative (i.e. smaller) PRE to 0 min POST-PAS change in the LICI ratio (see Supplementary Materials Figure S7). While exploratory, this suggests that sleepiness scores, which may reflect alertness during testing, could influence PAS-induced GABA_B_-mediated inhibitory responses and contribute to inter-individual variability.

### Associations between paired associative stimulation-induced long-intracortical inhibition increase and function

A significant negative association between PAS-induced increase in LICI and simple bilateral pegboard performance was evident ~6 weeks following injury, suggesting that greater modulation of GABA_B_-mediated inhibition is associated with better bilateral performance. Similar associations were evident ~3 weeks and ~ 12 weeks following burn injury, though they did not reach statistical significance, suggesting the association is strongest ~6 weeks following burn injury. As described above, greater baseline, resting state LICI 1–3 years following burn injury is associated with better motor performance [33]. While the current study showed no association between baseline LICI and motor performance in the subacute period, it is interesting to speculate whether those who show the greatest PAS-induced change in LICI, potentially reflecting the greatest capacity for plasticity of LICI circuits, show the best recovery of motor performance in the chronic period following burn injury. While randomized controlled trials are required to determine whether early intervention with non-invasive brain stimulation to modulate LICI circuits would improve functional recovery, newly approved “accelerated TMS” protocol for the treatment of depression offer a likely feasible approach within the subacute period following burn injury: accelerated TMS protocols involve delivering 10 sessions of rTMS daily for 5 days [92].

No significant associations between PAS-induced neuroplasticity and self-reported physical or mental health outcomes were evident, suggesting no relationship between capacity for LTP-like plasticity and self-reported general function in this cohort. Previous research showed an association between LTD-like plasticity (induced by spaced cTBS) and general health perception scores ~12 weeks following injury, suggesting those subacute burn patients with a greater plasticity response have the best recovery within this timeframe [41]. Together, these findings might suggest the distinct neurophysiological processes of LTP and LTD plasticity are differentially associated with self-report functional recovery [93].

### Limitations and future directions

While the current study provided the first comprehensive characterization of neurophysiological changes in the subacute period following burn injury, there are some limitations that should be considered when interpreting the findings.

First, the cohort size (*n =* 34) and predominance of non-severe burns limit statistical power to detect subtle effects and preclude definitive subgroup inference. Although the repeated-measures design and trial-level modeling used within-participant and within-session information, estimates for subgroup results should be interpreted cautiously and require replication in larger cohorts. The current findings apply to patients with non-severe burns, who represent the majority of burn injuries in the community [94]; future research is needed to determine whether similar neurophysiological and functional patterns occur after larger burns. Future research should also use stratified samples and analyses to investigate whether burn location or demographic factors, such as age, influence neuroplasticity responses following burn injury. Furthermore, medical history and health characteristics of burn patients might influence neurophysiological and functional patterns of recovery, and future research should record and include data such as presence of infection, medication use, and comorbidities in analyses. Future research using stratified samples should also undertake *a priori* sample size calculations (or simulation-based power analyses for mixed-effects models) to ensure adequate statistical power to detect clinically meaningful effects.

Second, although visits were scheduled according to the pre-specified protocol wherever possible, actual timing was constrained by participant availability and clinical factors, resulting in an unbalanced dataset (only 22 participants completed all three visits) and comparatively limited data near the boundaries of the observation window. We therefore focused inference on 23, 44, and 82 days post-injury, which corresponded to the highest testing density and clinically relevant milestones. While spline-based modeling can accommodate non-linearity, it cannot fully eliminate the possibility of residual bias if recovery slopes differ systematically between participants with different follow-up patterns, particularly near the edges where data were sparse. To mitigate this, models included participant-level random effects to account for within-subject dependence and between-subject heterogeneity. Nevertheless, estimated trajectories (especially near the boundaries) should be interpreted cautiously, and future studies with more balanced coverage across the recovery window will further strengthen inference about recovery trajectories.

Third, in the PAS protocol, a standard interval of 25 ms was used between the peripheral stimulus and the TMS to M1: this approach does not account for individual differences in conduction time and might result in suboptimal timing for PAS plasticity induction [95]. In addition, for three participants with wrist burns, the peripheral stimulating electrodes were placed on the palmar surface of the third digit on the most affected side, rather than over the median nerve at the wrist. This electrode placement stimulated the median nerve via the palmar digital branches [52] would have affected the precise timing of the PAS protocol, potentially affecting PAS-induced plasticity. Moreover, PAS was administered at various times of the day, which could have introduced variability in the PAS response due to circadian fluctuations [87, 96]. Participants also reported increased sleepiness following PAS, with the most pronounced changes occurring immediately post-PAS.

Fourth, several participants with hand burns noted that injury or dressings interfered with pegboard performance, introducing variability in motor scores unrelated to neurophysiological function. Separately, to ensure deficits on the most-affected side were reflected in the outcome, we emphasized bilateral pegboard performance in the main analyses; because subgrouping by most- versus least-affected hand would have been underpowered, we did not stratify results by combinations of burn side and handedness. Consequently, weak or null associations between intracortical inhibition and pegboard performance in the subacute period should be interpreted cautiously, acknowledging local injury-related constraints on hand use may have diluted relationships.

Fifth, while our primary aim was to characterize changes in neurophysiology and function during the subacute period following burn injury, we did not include a prospectively enrolled age- and sex-matched longitudinal non-injured comparison cohort. As a result, we cannot fully separate injury-related neurophysiological change from non-specific temporal influences (e.g. practice effects, maturational drift, diurnal/seasonal fluctuation). The additional cross-sectional analyses comparing a subsample of burn participants with non-injured controls would be strengthened by incorporating prospectively enrolled, age- and sex-matched longitudinal comparison groups where feasible, or by using intervention-based designs with appropriate prospective comparison groups and TMS outcomes assessed alongside functional measures.

Sixth, neurophysiological measures were restricted to the hand representation of M1 and potential changes in spinal excitability, inter-hemispheric connectivity, and cerebellar brain inhibition were not assessed. Furthermore, these measures were only assessed up to 12 weeks following burn injury, which limits inference about whether early inhibitory modulation predicts longer-term functional trajectories. Future research should include comprehensive physiological assessment (e.g. spinal, inter-hemispheric, and cerebellar measures) with longer follow-up to better understand associations between central nervous system changes and functional recovery trajectories.

Finally, both pre-injury and post-injury medications, including pain relief and antidepressants, which act on the central nervous system [97], as well as surgical treatment for the burn injury [98], may have influenced the observed findings. Future studies should record these and account for these potential confounders by stratifying participants based on pain or medication use or incorporating these factors as covariates in analyses.

## Conclusions

Subacute burn patients with non-severe injuries showed greatest improvements in motor performance and self-reported physical health from ~3 to ~6 weeks following injury. Changes in GABA_B_-mediated inhibition were evident in the first ~12 weeks following burn injury: baseline resting-state LICI inhibitory influence increased over the first 12-weeks following injury, and PAS induced an increase in LICI, which was greatest at ~6 weeks following injury and was significantly associated with bilateral motor performance. These findings suggest that burn patients who show stronger modulation of GABA_B_-mediated inhibition in response to PAS, potentially reflecting greater plasticity within these circuits, might have enhanced opportunity for motor recovery in the subacute phase following burn injury. While the current results must be replicated, they do provide promise that the excitability of GABA-B mediated inhibitory circuits, and the capacity for plasticity of these circuits, might be a predictor of functional recovery following burn injury. For example, quantifying capacity for neuroplasticity in LICI at the ~6 week clinical could identify patients who are likely to recover well (large neuroplasticity response) and those who are likely to recover less well (small neuroplasticity response). For those with a small neuroplasticity response, targeted non-invasive brain stimulation (such as rTMS or transcranial electrical stimulation) that modulated LICI circuits could be considered as an adjunct therapy to standard rehabilitation. Future research should comprehensively investigate GABA_B_-mediated inhibition as a marker of functional recovery and a target for non-invasive brain stimulation treatments following burn injury.

## Supplementary Material

Supplementary_materials_tkag034

Supplmentary_Material_tkag034
